# Efficacy and safety of nafamostat mesilate anticoagulation in blood purification treatment of critically ill patients: a systematic review and meta-analysis

**DOI:** 10.1080/0886022X.2022.2105233

**Published:** 2022-08-05

**Authors:** Yao Lin, Yiming Shao, Yuchun Liu, Ruoxuan Yang, Shuanglin Liao, Shuai Yang, Mingwei Xu, Junbing He

**Affiliations:** a Jieyang Medical Research Center, Jieyang People's Hospital, Jieyang, China; b The Intensive Care Unit, The Second Affiliated Hospital of Guangdong Medical University, Zhanjiang, China

**Keywords:** Nafamostat mesilate, blood purification, bleeding complication, mortality, COVID-19

## Abstract

**Background:**

Nafamostat mesilate (NM), a broad-spectrum and potent serine protease inhibitor, can be used as an anticoagulant during extracorporeal circulation, as well as a promising drug effective against coronavirus disease 2019 (COVID-19). We conducted a systematic meta-analysis to evaluate the safety and efficacy of NM administration in critically ill patients who underwent blood purification therapy (BPT).

**Methods:**

The Cochrane Library, Web of Science and PubMed were comprehensively searched from inception to August 20, 2021, for potential studies.

**Results:**

Four randomized controlled trials (RCTs) and seven observational studies with 2723 patients met the inclusion criteria. The meta-analysis demonstrated that conventional therapy (CT) significantly increased hospital mortality compared with NM administration (RR = 1.25, *p* = 0.0007). In subgroup analyses, the in-hospital mortality of the NM group was significantly lower than that of the anticoagulant-free (NA) group (RR = 1.31, *p* = 0.002). The CT interventions markedly elevated the risk ratio of bleeding complications by 45% (RR = 1.45, *p* = 0.010) compared with NM interventions. In another subgroup analysis, NM used exhibited a significantly lower risk of bleeding complications than those of the low-molecular-weight heparin (LMWH) used (RR = 4.58, *p* = 0.020). The filter lifespan was decreased significantly (MD = −10.59, *p* < 0.0001) in the NA groups compared with the NM groups. Due to the poor quality of the included RCTs, these results should be interpreted with caution.

**Conclusion:**

Given the better survival outcomes, lower risk of bleeding, NM anticoagulation seems to be a safe and efficient approach for BPT patients and could yield a favorable filter lifespan. More multi-center RCTs with large samples are required for further validation of this study.

## Introduction

Since December 2019, the coronavirus disease 2019 (COVID-19) outbreak and pandemic caused by severe acute respiratory syndrome coronavirus 2 (SARS-CoV-2) have posed a great challenge to human health and social stability [1]. Although most COVID-19 patients present with mild or no symptoms, some of these cases may develop septic shock and multiple organ dysfunction syndrome (MODS) that require intensive care unit (ICU) treatment and extracorporeal organ support [2]. In the absence of specific medicines and vaccines, extracorporeal blood purification therapies (BPTs) could afford patients a survival benefit following severe COVID-19 through clearance of proinflammatory mediators and multiorgan system life support [3–5].

BPTs encompass various techniques including high-volume hemofiltration (HVHF), high-adsorption hemofiltration, coupled plasma filtration adsorption (CPFA), hemoadsorption, plasma exchange, hemoperfusion, and high-cutoff (HCO) hemodialysis/hemofiltration [6]. During extracorporeal blood purification, blood components contact the foreign surface of the circuit, which may cause coagulation cascades and result in clotting in the catheter and filter. Anticoagulants are commonly used to prevent such events. Unfractionated heparin (UFH) is the most frequently used anticoagulant, but it can be a double-edged sword, as it may lead to heparin-induced thrombocytopenia (HIT), a life-threatening complication associated with an increased risk of bleeding [7,8]. Accumulated evidence has revealed that thrombocytopenia is prevalent in acute COVID-19-infected patients at admission [9]. In addition, a recent study also found that 11.8% percent of COVID-19 patients suffered delayed-phase thrombocytopenia, which occurred 14 days after the onset of COVID-19 symptoms [10]. This combination of renal dysfunction and viral infection in patients who need blood purification makes the risk of bleeding more difficult to predict. Thus, the first crucial step for BPT patients is the selection of an appropriate anticoagulant protocol.

Nafamostat mesilate (NM), a broad-spectrum and potent serine protease inhibitor, has attracted much attention for its ability to decrease SARS-CoV-2 S protein-mediated membrane fusion and prevent viral infection [11,12]. As an anticoagulant, NM is not only used to reverse the coagulopathy of patients with disseminated intravascular coagulation (DIC) but also used for extracorporeal circulation anticoagulation [13,14]. The choice of anticoagulant depends on the condition of the hemostatic system. Since activated coagulation, decreased procoagulatory potential, or, even worse, a combination of both might exist in severely ill COVID-19 patients, the ideal anticoagulant for these particular patients should be resistant to filtration coagulation and have a lower risk of bleeding complications [10,15]. NM has been developed for nearly 30 years (mainly in Japan), and research has indicated that the greatest advantage of NM is its extension of the lifespan of the filters used during the hemodialysis process for patients with a high risk of bleeding [16,17]. Hence, NM might be more suitable for critically ill patients who underwent BPT in the current phase of the COVID-19 pandemic.

However, there is still a lack of systematic evaluation of the efficacy, safety and adverse complications of NM in critically ill patients during blood purification. Therefore, we conducted this systematic meta-analysis, for the first time, to compare the bleeding risk, filter lifespan and in-hospital mortality rate between NM and conventional therapy (CT), such as UFH, low-molecular-weight heparin (LMWH), citrate (C) and anticoagulant-free (NA), to evaluate the safety and efficacy of NM administration in critically ill patients who underwent blood purification in the ICU.

## Materials and methods

### Protocol and registration

This meta-analysis was performed using the Cochrane review methodology in accordance with the Preferred Reporting Items for Systematic Reviews and Meta-analyses (PRISMA) guidelines (Additional File 1) [18]. The protocol for this systematic review was registered with INPLASY (202180118) and is available in full at inplasy.com (https://doi.org/10.37766/inplasy2021.8.0118).

### PICO question

The patient population, intervention, comparison, and outcome (PICO) framework were used to formulate the following questions:

1. For BPT patients, does NM more effective in extending filter lifespan than conventional anticoagulant therapy?
2. For BPT patients, does the administration of NM result in a lower risk of bleeding complications than administration of conventional anticoagulant therapy?
3. For BPT patients, does NM provide better survival outcomes than conventional anticoagulant therapy?

### Search methodology

A comprehensive literature search of the Cochrane Library, Web of Science and MEDLINE databases (*via* the PubMed search engine) was performed to identify studies meeting the inclusion criteria from inception to August 20, 2021. This systematic review identified all publications presenting comparisons of NM. Eligible studies were identified using the following text words or Medical Subject Headings (MeSH): Nafamostat, Nafamostat mediate, Nafamostat Dimethanesulfonate, FUT-175, Futhan, renal replacement, blood purification therapy, Hemopurification, hemoperfusion, hemoadsorption, plasmafiltration, plasmafiltration and renal replacement therapy [MeSH Terms]. The search strategy and details of the query are presented in Additional File 2. In addition, the reference lists of retrieved studies and review articles were further manually searched for additional publications. No language restriction was used.

### Study selection criteria and data extraction

Two reviewers (Lin Yao and Yi-Ming Shao) independently performed an initial eligibility screen of all article types, titles, and abstracts. Publications that specifically referred to the administration of NM in patients receiving BPT were collected for further review. Next, full-text reviews were conducted independently, and studies meeting the following criteria were candidates for inclusion: 1) observational cohort and/or randomized/quasi-randomized clinical trial design; 2) patients with various levels of organ dysfunction; and 3) all patients underwent BPT, and the conventional anticoagulant strategy was compared with NM. The exclusion criteria were as follows: 1) duplicate studies; and 2) review articles, case reports, letters, meta-analyses, animal experimental studies, and so on. Data extraction was carried out by two of the authors (Ruoxuan Yang and Shuanglin Liao) independently, and any disagreement between the two authors was resolved according to the assessment of a third author or discussion. The data extracted included the study ID, first author and year of publication, type of study, sample size, age of participants, etiology and diagnosis, details of BPT and endpoint.

### Assessment of methodological quality

Randomized studies were appraised using the Cochrane Collaboration Risk of Bias Tool [19]. The following characteristics will be evaluated: 1) sequence generation; 2) allocation concealment; 3) blinding; 4) incomplete outcome data; 5) selective outcome reporting; and 6) other potential threats to validity. In addition, observational studies (prospective and retrospective cohorts) were evaluated by using a modified version of the Quality Assessment Tool for Observational Cohort and Cross-Sectional Studies published by the National Institutes of Health [20]. Criteria items were evaluated for each study as follows: 1) research question; 2) study population; 3) uniform eligibility criteria; 4) sample size justification; 5) timing of exposure assessment; 6) sufficient time frame to see an effect; 7) different levels of the exposure of interest; 8) exposure assessed prior to outcome measurement; 9) outcome measures; 10) blinding; and 11) statistical analyses. Quality assessments were undertaken independently by Lin Yao and Yi-Ming Shao, and any disagreements were resolved by consensus with a third author (Yu-Chun Liu).

### Statistical analysis

The data were extracted and assessed by using Review Manager software (version 5.3, The Nordic Cochrane Center, Cochrane Collaboration) and STATA statistical software (version 12.0) to make the outcome assessment more comprehensive. Estimated effects were reported as RRs with 95% CIs for dichotomous outcomes and mean differences (MDs) with 95% CIs for continuous outcomes. Heterogeneity was assessed for each pooled summary estimate using Cochran’s Q statistic and the *I*^2^ statistic, and the thresholds for high, moderate and low heterogeneity were set at >75%, 25–75% and <25%, respectively [21]. A random-effects model was applied to pool the results across the studies for which there was formal evidence of statistical heterogeneity (significance for *I*^2^ >50%). For studies with lower levels of statistical heterogeneity, a fixed-effect model was employed to pool the outcomes. We assessed the potential publication bias of the meta-analysis by using Begg’s and Egger’s tests. To test the robustness of the pooled estimates, we evaluated whether fixed-effects models and random-effects would bring about the same outcome. The stability of the results was also further confirmed by using leave-one-out sensitivity analyses. All statistical tests were two-tailed, and *p* < 0.05 was used to indicate statistical significance.

### Confidence in evidence

We evaluated the confidence in the evidence using the Grading of Recommendations Assessment, Development, and Evaluation (GRADE) approach, which classified evidence as high, moderate, low, or very low certainty for each outcome [22].

## Results

### Study selection

**Figure 1.**
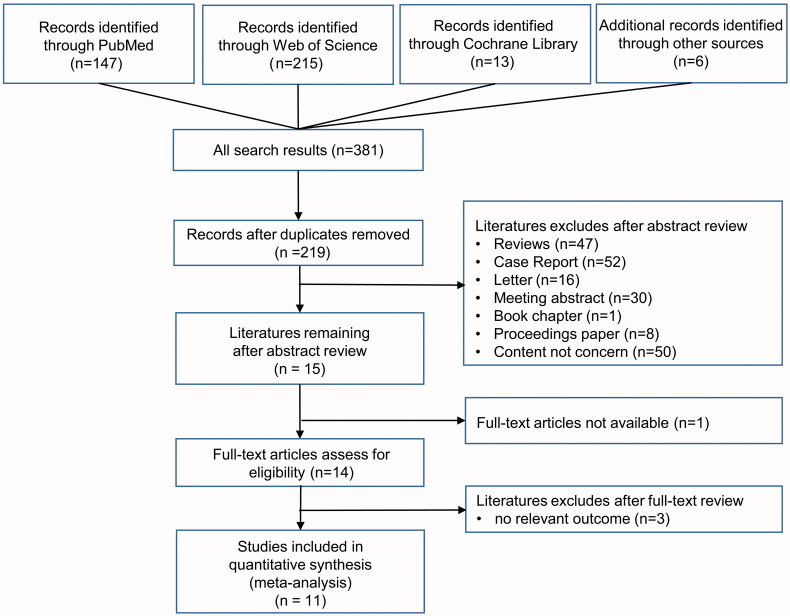
Study inclusion flow chart.

The study inclusion flow chart is shown in Figure 1. The number of studies identified from the Cochrane Library, Web of Science and PubMed databases was 13, 215 and 147, respectively. An additional 6 studies were manually identified from the reference lists of the included publications. Among these identified references, 162 duplicate publications were identified and excluded from this systematic meta-analysis. Furthermore, 204 studies were excluded based on our inclusion and exclusion criteria after a title and abstract screen, resulting in 15 publications that underwent full-text review. In addition, the full text was not available in 1 study [23], and 3 of these remaining publications were removed due to an uncorrelated outcome [24–26]. Ultimately, 7 observational studies and 4 randomized controlled trials with 2723 patients were enrolled in this systematic meta-analysis [17,27–36].

### Study characteristics

The characteristics of the 11 enrolled studies are presented in Table 1. Among these studies, 4 papers were randomized trials (comparison of NM with NA) [17,27–29], 1 paper was a prospective cohort study [31], and 6 papers were retrospective cohort studies [30,32–36]. Most of these studies were published after 2010 (82%), and only one study [31] was a multicenter trial. Deteriorated renal function was the main cause of BPT in nine studies [17,27,29,31–36], whereas the other two studies [28,30] did not report etiology. The detailed protocols of BPT in these studies are presented in Table 2. Continuous renal replacement therapy (CRRT) was used as the most common modality for BPT in 10 studies [17,27–35]. The CRRT mode was continuous venovenous hemofiltration (CVVH) in 3 studies [17,27,33], continuous venovenous hemodiafiltration (CVVHDF) in 3 studies [28,32,34], and a combination of CVVH and CVVHDF in 2 studies [30,31]. RRT, polymyxin B-immobilized hemoperfusion (PMX-HP), and/or plasma exchange (PE) in 1 study [36]. However, the remaining 2 studies did not report the CRRT mode [28,35]. All 11 studies compared data across groups divided by different anticoagulant strategies, among which 5 studies compared NA with NM [17,27–29,33], 2 studies compared NM with UFH and NA [33,34], 1 study compared NM with UFH [35] and the other 1 study compared NM with UFH and LMWH [30]. The remaining 2 studies compared NM with more than two anticoagulant schemes [31,35], and one of them did not classify conventional anticoagulant regimens [36]. The loading dose of NM used in most studies was 20 mg/h [17,28,29,35]. Two studies used low doses of NM, 12.5 mg/h and 10 mg/h [27,32]. NM was administered at the required dose per kilogram per hour (0.1 mg/kg/h and 0.25 mg/kg/h) in two other studies [30,34]. Maintenance doses of NM were adjusted according to each patient’s status and ranged from 5 mg/h to 30 mg/h in most studies [17,27–29,32,33].

**Table 1.** Characteristics of the included studies.

Study	Country	Design	Arms	Age (year)	Sex	Setting	Reason to start BPT	APACHE II	Platelet (×1,000/uL)	aPTT (sec)	Endpoint
Park et al. [17]	Korea	RCT	NM	61.25 ± 18.23	F8/M12	ICU	ARF due to sepsis, surgical and others	17.35 ± 5.13 for NM	152.25 ± 117.99 for NM	58.19 ± 32.95 for NM	Mortality
			NA	59.00 ± 10.29	F12/M11			15.52 ± 3.96 for NA	158.83 ± 98.81 for NA	50.59 ± 26.05 for NA	
Kim et al. [27]	Korea	RCT	NM	58.8 ± 14.60	F9/M8	ICU	ARF	NR	224.4 ± 136.2 for NM	30.1 ± 5.7 for NM	Bleeding complication
			NA	57.1 ± 16.80	F8/M10				187.3 ± 66.2 for NA	30.0 ± 4.8 for NA	
Lee et al. [28]	Korea	RCT	NM	52.97 ± 13.94	F12/M24	ICU	NR	26.72 ± 5.26 for NM	57.44 ± 40.05 for NM	NR	Mortality; distribution of filter lifespan; bleeding complication
			NA	57.54 ± 13.04	F17/M20			26.84 ± 6.00 for NA	90.92 ± 97.39 for NA		
Choi et al. [29]	Korea	RCT	NM	63.6 ± 11.5	F10/M21	ICU	AKI due to sepsis, Ischemia, toxin hypovolemia and cardiac failure	23.5 ± 6.2 for NM	115.5 ± 75.9 for NM	52.5 ± 39.3 for NM	Filter lifespan; survival rate
			NA	58.6 ± 18.0	F11/M15			25.9 ± 8.4 for NA	77.7 ± 65.6 for NA	46.3 ± 28.2 for NA	
Ohtake et al. [30]	Japan	Retrospective cohort	NM	NR	NR	ICU	NR	NR	NR	NR	Bleeding complication
			UFH								
Uchino et al. [31]	23 countries	Prospective observation	NM	NR	NR	ICU	ARF due to sepsis, major surgery, low cardiac output, Hypovolemia and others	NR	NR	NR	Bleeding complication; mortality
			UFH								
			C								
Baek et al. [32]	Korea	Retrospective cohort	NM	59.3 ± 15.9	F22/M40	ICU	AKI due to sepsis, cardiac failure, hepatorenal syndrome and others	NR	NR	NR	Bleeding complication; mortality
			NA	55.5 ± 12.7	F67/M114						
Hwang et al. [33]	Korea	Retrospective cohort	NM	65.2 ± 11.0	F9/M16	ICU	Renal, Cardiac, Neurological, Sepsis and others	25.6 ± 7.1 for NM	136.9 ± 108.4 for NM	54.9 ± 25.3 for NM	Filter lifespan; bleeding complication; survival rate
			UFH	69.5 ± 13.0	F31/M25			24.3 ± 5.8 for UFH	180.0 ± 93.1 for UFH	41.3 ± 13.4 for UFH	
			NA	66.3 ± 15.0	F54/M77			24.9 ± 6.1 for NA	164.6 ± 139.1 for NA	50.7 ± 21.2 for NA	
			UFH-S	73.6 ± 10.1	F5/M5			26.2 ± 6.2 for UFH-S	200.3 ± 95.4 for UFH-S	45.9 ± 19.4 for UFH-S	
Lee et al. [34]	Korea	Retrospective cohort	NM	6.9 ± 5.6	F6/M9	ICU	AKI due to sepsis, TLS/Rhabdomyolysis and others	NR	43.2 ± 21.6 for NM	50.8 ± 23.5 for NM	Filter lifespan; bleeding events; survival rate
			UFH	9.2 ± 9.8	F2/M4				328.0 ± 185.3 for UFH	45.2 ± 11.2 for UFH	
			NA	9.2 ± 6.4	F7/M12				36.7 ± 21.6 for NA	73.9 ± 74.7 for NA	
Makino et al. [35]	Japan	Retrospective cohort	NM	67–82	F27/M49	ICU	Renal and others	17–20 for NM	62–148 for NM	28–38 for NM	Bleeding complication; filter lifespan
			UFH	70–83	F3/M22			14–21 for UFH	65–205 for UFH	30–49 for UFH	
Kamijo et al. [36]	Japan	Retrospective cohort	NM	62–78	F495/M510	ICU	Sepsis (renal and/or non-renal indication)	21–32 for NM	53–165 for NM	NR	Bleeding complication; mortality
			CT	60–79	F170/M240			17–28 for CT	59–178 for CT		

Abbreviations: RCT, Randomized controlled trial; NM, nafamostat mesilate; UFH, unfractionated heparin; LMWH, low molecular weight heparin; C, citrate; NA, anticoagulant-free; CT, conventional anticoagulant therapy; ICU, intensive care unit; AKI, acute kidney injury; ARF, acute renal failure; APACHE, acute physiology and chronic health evaluation; F, female; M, male; NR, not reported.

**Table 2.** Characteristics of BPT and main outcomes.

Study	Modality	Arms	Patients, *N*	Blood flow rate	Loading dose	Maintenance doses	Mean filter lifespan	Bleeding complication *N* (%)	Hospital mortality *N* (%)
Park et al. [17]^#^	CVVH	NM	20	100–150 ml/min	20 mg/h	10–20 mg/h	28.73 ± 12.67 hours	0 (0.0)	NR
		NA	23		–	–	16.34 ± 7.86 hours	0 (0.0)	
Kim et al. [27]^#^	CVVH	NM	17	230–300 ml/min	12.5 mg/h	12.5 mg/h	NR	2 (11.8)	NR
		NA	18		–	–		2 (11.1)	
Lee et al. [28]^#^	CRRT	NM	36	130–200 ml/min	20 mg/h	10–30 mg/h	26.63 ± 21.14 hours 22.70 ± 20.67 hours	5 (15.6)	24 (75.0)
		NA	37		–	–		5 (17.8)	20 (74.1)
Choi et al. [29]^#^	CVVHDF	NM	31	150–200 ml/min	20 mg/h	10–30 mg/h	31.7 ± 24.1 hours	2 (6.5)	17 (47.2)
		NA	24		–	–	19.5 ± 14.9 hours	0 (0.0)	19 (52.8)
Ohtake et al. [30]	CCVH/CVVHDF	NM	23	NR	0.1 mg/kg/h	0.1 mg/kg/h	NR	1 (4.0)	NR
		UFH	12		–	–		8 (67.0)	
		LMWH	17		–	–		5 (29.0)	
Uchino et al. [31]	CVVH/CVVHDF /CAVHD	NM	61	150–200 ml/min	NR	NR	NR	2 (3.3)	NR
		UFH	429					10 (2.3)	
		LMWH	44					5 (11.4)	
		C	99					2 (2.0)	
Baek et al. [32]^#^	CVVHDF	NM	62	120–150 ml/min	10 mg/h	5–20 mg/h	NR	NR	26 (41.9)
		NA	181		–	–			117 (64.6)
Hwang et al. [33]^#^	CVVH	NM	25	100–150 ml/min	NR	10–30 mg/h	NR	NR	15 (60.0)
		UFH	56			1U–20 U/kg/h			29 (51.8)
		NA	131			–			84 (64.1)
		UFH-S	10			–			5 (50.0)
Lee et al. [34]^#^	CVVHDF	NM	25	3–5 ml/kg/min	0.25 mg/kg/h	0.125–0.5 mg/kg/h	NR	0 (0.0)	8 (40.0)
		UFH	6		20 U/kg/h	10 U/kg/h		0 (0.0)	0 (0.0)
		NA	19		–	–		0 (0.0)	11 (58.9)
Makino et al. [35]*	CRRT	NM	76	NR	20 mg/h	NR	NR	5 (6.6)	NR
		UFH	25		400 IU/h			4 (16.0)	
Kamijo et al. [36]*	RRT/PMX-HP/PE	NM	805	NR	NR	NR	NR	129 (16.0)	343 (42.6)
		CT	411					69 (16.8)	158 (38.4)

Abbreviations: CRRT, continuous renalreplacement therapy; RRT, renal replacement therapy; CVVH, continuous venovenous hemofiltration; CVVHD, continuous venovenous hemodialysis; CVVHDF, continuous venovenous hemodiafiltration; CAVHD, continuous arteriovenous hemodialysis; PMX-DH, polymyxin B-immobilized hemoperfusion; PE, plasma exchange; NM, nafamostat mesilate; UFH, unfractionated heparin; LMWH, low molecular weight heparin; C, citrate; NA, anticoagulant-free; CT, conventional anticoagulant therapy; N, number of patients; NR, not reported; -, not application; *adjusted outcomes; ^#^baseline balance.

### Quality evaluation

**Figure 2.**
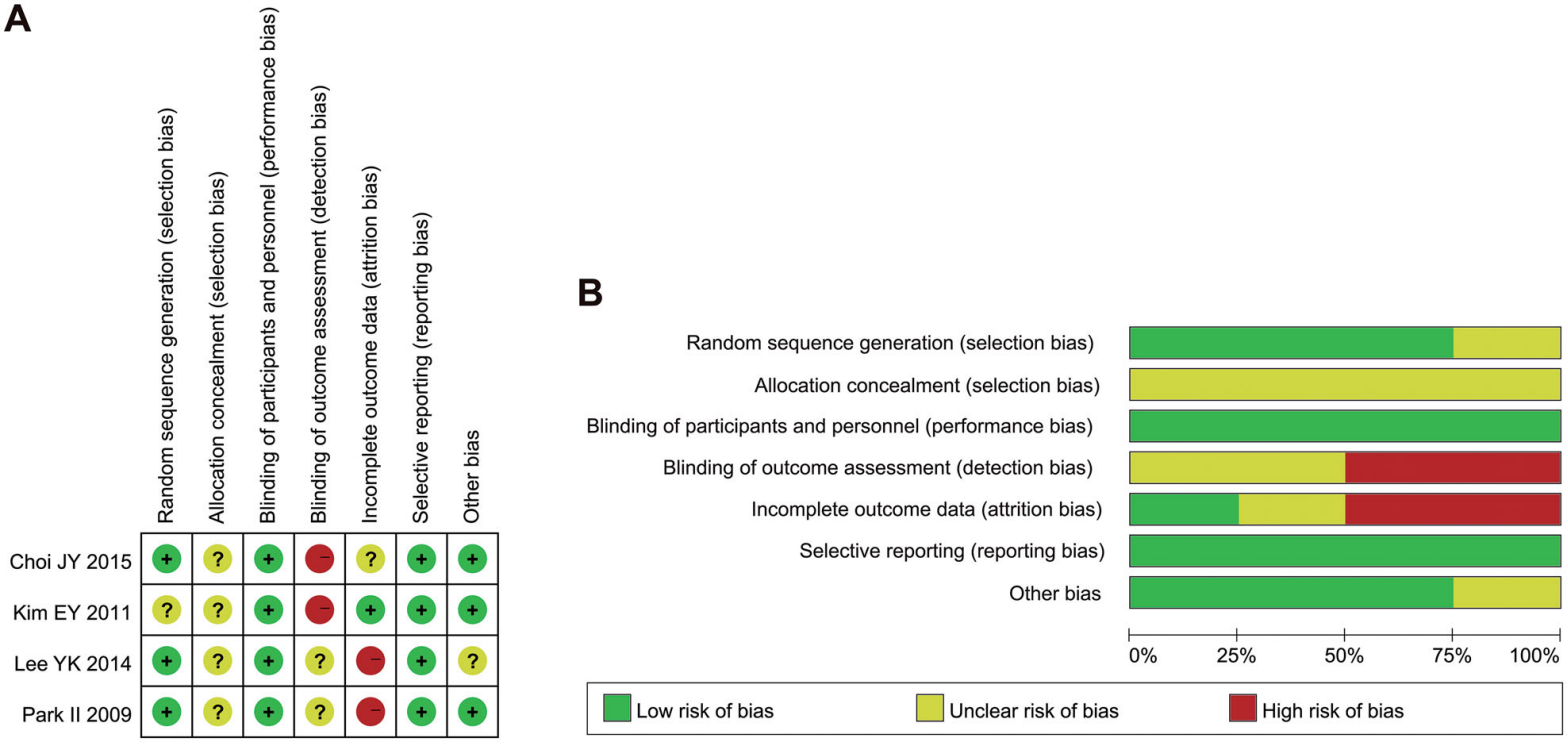
Risk of bias and summary of the risk of bias of 4 enrolled RCTs.

The methodological quality of the 4 enrolled randomized controlled trials is presented in Figure 2, and none of the studies described allocation concealment. Because the two interventions were significantly different, blinding of participants and personnel was not practical, but lack of blinding did not produce significant bias; thus this domain was not considered for the study-level assessment. Blinding of outcome assessment was not performed in two studies [27,29], and insufficient information was available to permit judgment of another two studies [17,28]. One study [28] was supported by an NM manufacturer (SK Chemical); therefore, the other biases were unclear. Two studies [17,28] had a high risk of incomplete outcomes since more than 10% of patients in these two studies dropped out after allocation. We considered all four RCTs to be at high risk of bias in one or more criteria and rated them as of 'poor overall quality’. In addition, none of the cohort studies fulfilled all quality indicators, and the quality assessment scores ranged from 4 to 10 (out of a possible score of 14; Additional File 3). The most common methodological limitations that existed in the enrolled studies were the lack of blinding of outcome assessors [30,32–36], sample size justification [30–36] and different levels of exposure as related to the outcome [30,32–36].

### Bleeding complication

**Figure 3.**
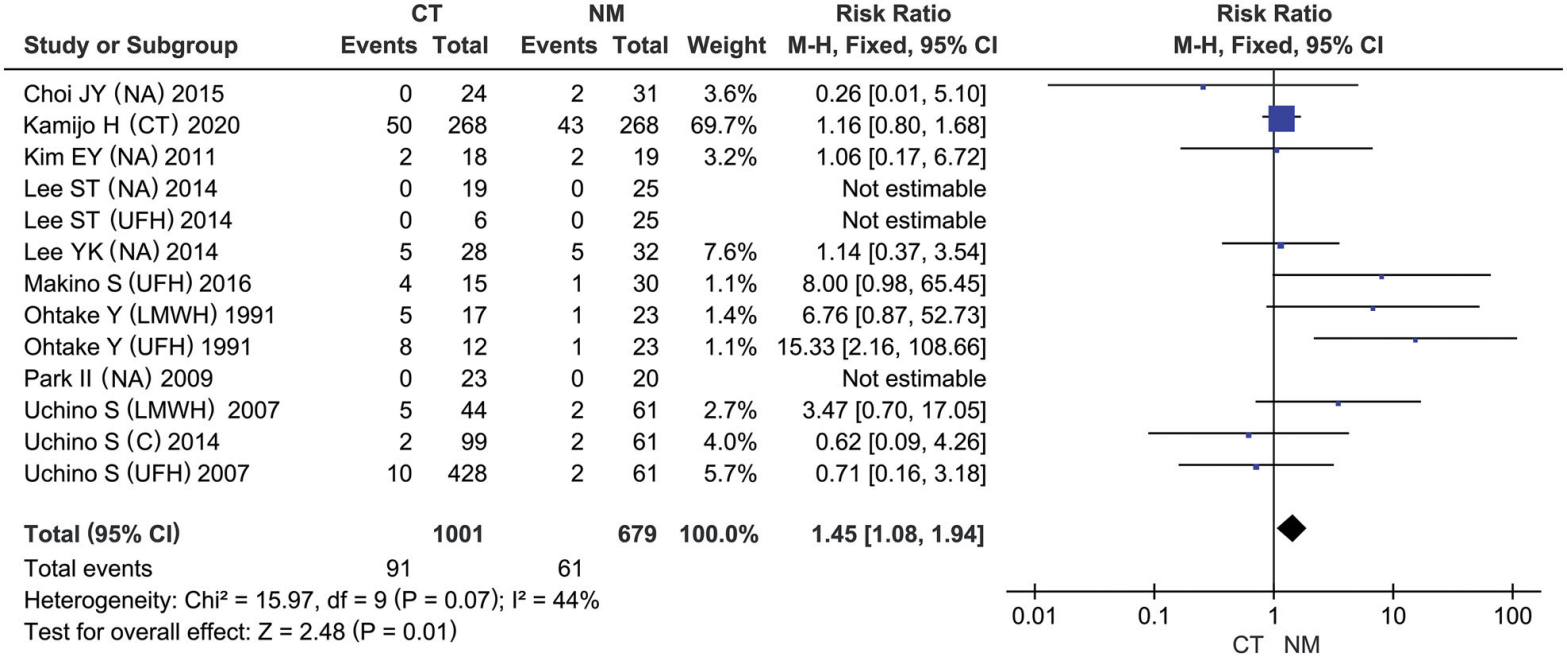
Forest plots of comparisons: CT versus NM; Outcomes: bleeding complications.

For the assessment of hemorrhage risk, bleeding complications were interpreted using the same standard: patients who experienced blood loss from a ruptured vessel were said to have bleeding complications. As presented in Figure 3, the number of patients with bleeding complications was reported in 9 of the included articles [17,27–31,34–36], with moderate heterogeneity (*I*^2^ = 44%, *p* = 0.07). A fixed-effects model showed that the conventional therapy (CT) group exhibited a significantly higher risk ratio of bleeding complications by 45% than the NM group (RR = 1.45, 95% CI: [1.08, 1.94], *p* = 0.01). However, when using a random-effects model to pool the results, the risk of bleeding complications in the conventional therapy group was observed to be higher than that in the NM group, with no statistical significance (Table 3, pooled RR =1.70, 95% CI: [0.91, 3.16], *p* = 0.09). Thus, it was necessary to conduct a subgroup analysis to improve the reliability of these results.

**Table 3.** Meta-analysis of the effect of anticoagulant interventions in patients under BPT.

Outcomes or subgroup analysis	Study reference number	Statistical method	Risk ratio or mean difference (95% CI)	*P*	*I*^2^ value (%)	Certainty of the evidence (GRADE)
Main outcomes: the effect of CT versus NM on bleeding complication						
Bleeding complication	[17,27–31,34–36]	M-H, fixed	RR = 1.45, CI: [1.08, 1.94]	0.01	44	⊕^⊝⊝⊝^
		M-H, random	RR = 1.70, CI: [0.91, 3.16]	0.09	44	Very low^a^
Subgroup analysis: the relationship between conventional anticoagulant interventions classification and bleeding complication						
NA	[17,27–29]	M-H, fixed	RR = 0.90, CI: [0.37, 2.22]	0.67	0	⊕^⊝⊝⊝^; Very low^a^
		M-H, random	RR = 0.97, CI: [0.37, 2.44]	0.95	0	
UFH	[30,31,34,35]	M-H, fixed	RR = 3.78, CI: [1.63, 8.76]	0.002	72	⊕^⊝⊝⊝^; Very low^a,b,c^
		M-H, random	RR = 4.04, CI: [0.54,30.52]	0.18	72	
LMWH	[30,31]	M-H, fixed	RR = 4.58, CI: [1.32, 15.91]	0.02	0	⊕^⊝⊝⊝^; Very low^b^
		M-H, random	RR = 4.46, CI: [1.27, 15.69]	0.02	0	
C	[30]	M-H, fixed	RR = 0.62, CI: [0.09, 4.26]	0.62	NR	⊕⊕^⊝⊝^; Low
		M-H, random	RR = 0.62, CI: [0.09, 4.26]	0.62	NR	
Unclassified	[36]	M-H, fixed	RR = 1.16, CI: [0.80, 1.68]	0.43	NR	⊕⊕^⊝⊝^; Low
		M-H, random	RR = 1.16, CI: [0.80, 1.68]	0.43	NR	
Main outcomes: the effect of CT versus NM on mortality						
In-hospital mortality	[28,29,30–34,36]	M-H, fixed	RR = 1.25, CI: [1.10, 1.43]	0.0007	42	⊕^⊝⊝⊝^; Very low^a,b,d^
		M-H, random	RR = 1.19, CI: [1.00, 1.42]	0.05	42	
Subgroup analysis: the relationship between conventional anticoagulant interventions classification and mortality						
NA	[28,29,32–34]	M-H, fixed	RR = 1.31, CI: [1.31, 1.55]	0.002	48	⊕^⊝⊝⊝^; Very low^b^
		M-H, random	RR = 1.26, CI: [1.00, 1.59]	0.05	48	
UFH	[33,34]	M-H, fixed	RR = 0.78, CI: [0.55, 1.13]	0.19	0	⊕^⊝⊝⊝^; Very low^a^
		M-H, random	RR = 0.84, CI: [0.59, 1.19]	0.32	0	
Unclassified	[36]	M-H, fixed	RR = 1.37, CI: [1.08, 1.73]	0.009	NR	⊕⊕^⊝⊝^; Low
		M-H, random	RR = 1.37, CI: [1.08, 1.73]	0.009	NR	
Secondary outcomes: the effect of CT versus NM on hemofilter lifespan						
Hemofilter lifespan	[17,28,29]	M-H, fixed	MD = −10.59, CI: [−15.45, −5.72]	<0.0001	0	⊕⊕^⊝⊝^; Low^a,b^
		M-H, random	MD = −10.59, CI: [−15.45, −5.72]	<0.0001	0	

**Abbreviation:** NR, not reported; M-H, Mantel-Haenszel; IV, Inverse Variance. NM, nafamostat mesilate; UFH, unfractionated heparin; LMWH, low molecular weight heparin; C, citrate; NA, anticoagulant-free; CT, conventional anticoagulant therapy.

^a^Downgraded one level due to imprecision (defined as wide confidence intervals including no effect and/or low overall sample size).

^b^Downgraded one level due to high risk of bias (incomplete outcome data).

^c^Downgraded one level due to large heterogeneity between studies.

^d^Downgraded one level due to serious inconsistency: point estimates varied widely.

**Figure 4.**
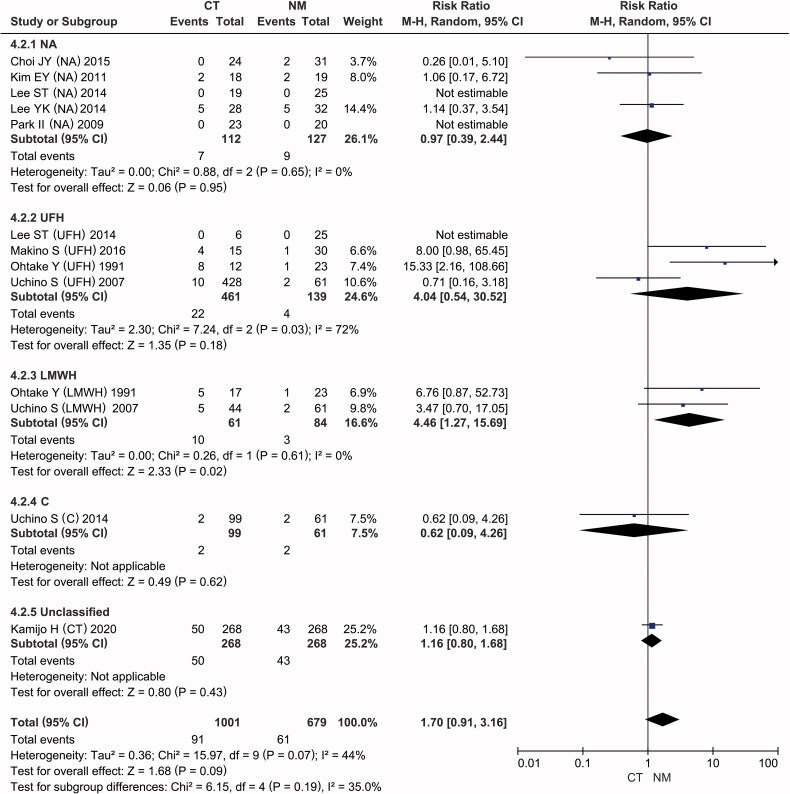
Forest plots of comparisons: subgroups of CT versus NM; Outcomes: bleeding complications.

Another subgroup analysis was conducted according to the anticoagulant used in the conventional therapy group. Four subgroups were identified: the NA, UFH, citrate and LMWH groups. As presented in Figure 4, the data showed that LMWH significantly increased the risk of bleeding complications in hemodialysis patients compared to NM (RR = 4.46, 95% CI: [1.27, 15.69], *p* = 0.02), with no intertrial heterogeneity (*I*^2^ = 0%, *p* = 0.61). In addition, there was no significant difference in the incidence of bleeding between the NA and NM groups (RR = 0.97, 95% CI: [0.39, 2.44], *p* = 0.95), without intertrial heterogeneity (*I*^2^ = 0%, *p* = 0.65). In light of the high heterogeneity (*I*^2^ = 72%, *p* = 0.03), the results of the UFH subgroup should be interpreted with caution (pooled RR = 4.04, 95% CI: [0.54, 30.52], *p* = 0.18).

### Mortality

**Figure 5.**
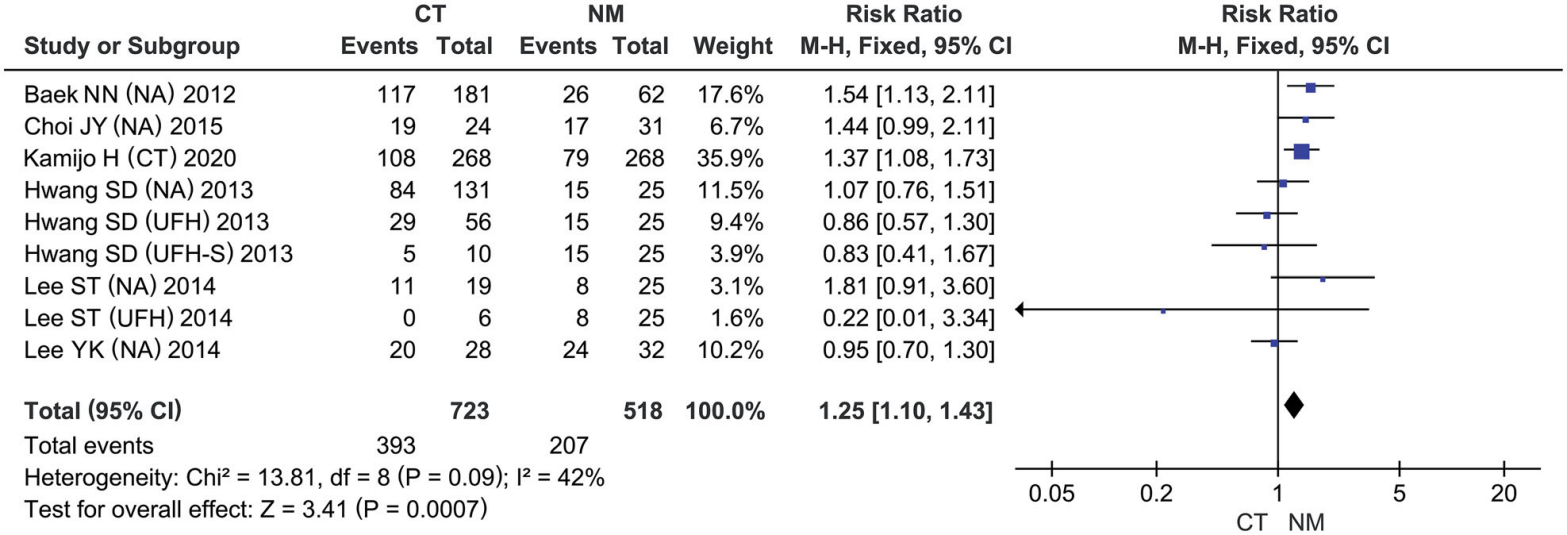
Forest plots of comparisons: CT versus NM; Outcomes: In-hospital mortality.

The RR for mortality is shown in Figure 5 and Table 3. In-hospital mortality was reported for six studies [28,29,32–34,36], with moderate heterogeneity (*I*^2^ = 42%, *p* = 0.09). Meta-analysis using a fixed-effects model demonstrated that CT interventions significantly increased hospital mortality compared with NM use (RR = 1.25, 95% CI: [1.10, 1.43], *p* = 0.0007), and the difference between the estimates was of borderline significance (*p* = 0.05) under a random-effects model.

**Figure 6.**
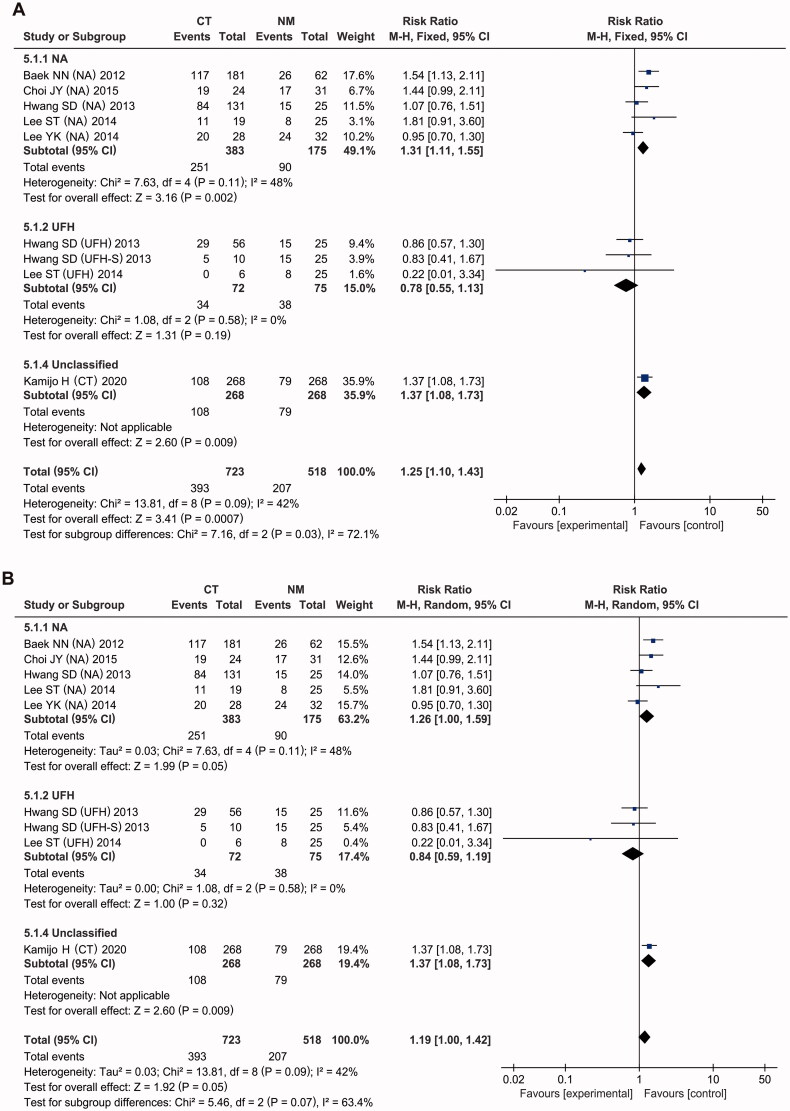
Forest plots of comparisons: subgroups of CT versus NM. Outcomes: In-hospital mortality under the fixed-effect model (A) and under the random-effect model (B).

Subgroup analysis was performed according to the type of anticoagulant used in the CT group. As presented in Figure 6 and Table 3, compared with that in the NM group, the risk of in-hospital mortality in the NA group was significantly higher in the meta-analysis under the fixed-effect model (RR = 1.31, 95% CI: [1.31, 1.55], *p* = 0.002), with moderate heterogeneity (*I*^2^ = 48, *p* = 0.11), but the increased risk had borderline significance under the random-effect model (pooled RR = 1.26, 95% CI: [1.00, 1.59], *p* = 0.05). Both the fixed-effect (RR = 0.84, 95% CI: [0.59, 1.19], *p* = 0.32) and random-effect model meta-analyses (RR = 0.78, 95% CI: [0.55, 1.13], *p* = 0.19) showed no significant effects of supplementation with UFH vs. NM on mortality in the included trials, without intertrial heterogeneity (*I*^2^ = 0%, *p* = 0.58).

### Hemofilter lifespan

**Figure 7.**
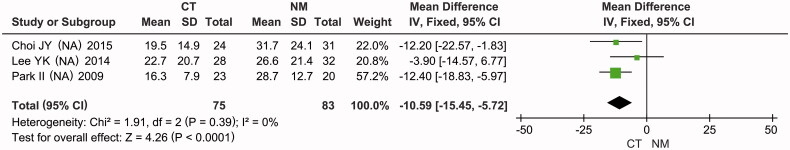
Forest plots of comparisons: CT versus NM. Outcomes: hemofilter lifespan.

The hemofilter lifespan was documented as the time until the new filter exchange. Data on hemofilter lifespan are presented as the mean ± SD and were available in 3 studies [17,28,29] without significant heterogeneity (Figure 7; *I*^2^ = 0%, *p* = 0.39). The mean filter lifespan ranged from 26.6 to 31.7 h in the NM groups and 16.3 to 22.7 h in the NA groups. Since no significant heterogeneity was observed, a random-effects model was used to calculate the pooled results. Compared with the NM group, the conventional treatment group showed a significantly decreased hemofilter lifespan (Figure 7 and Table 3, the pooled difference in mean = −10.59, 95% CI: [−15.45, −5.72], *Z* = 4.26, *p* < 0.0001). In addition, the pooled results of hemofilter lifespan did not change significantly under a fixed-effect model.

### Sensitivity analysis and publication bias

**Figure 8.**
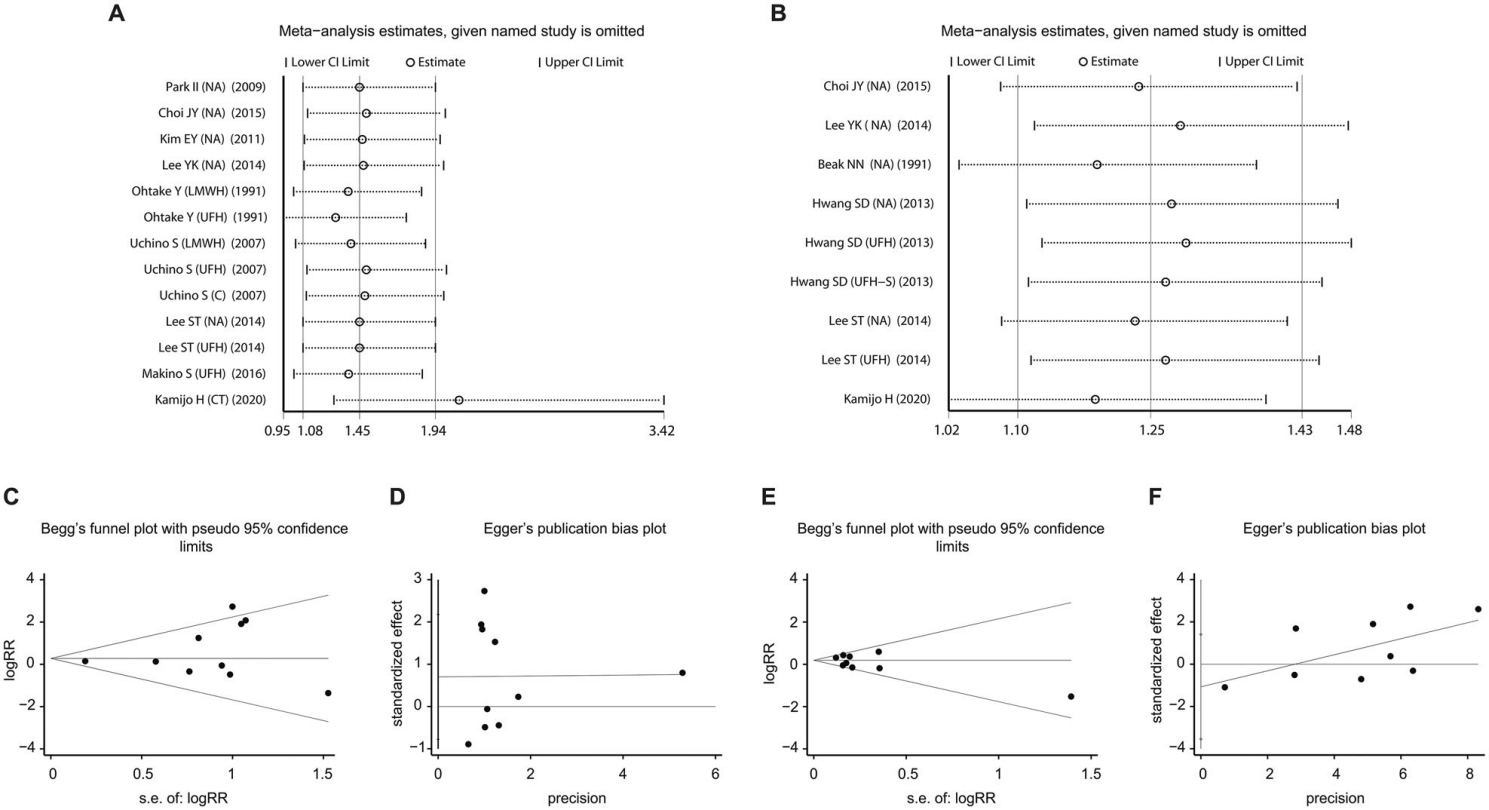
Sensitivity analysis shows the meta-analysis has satisfactory stability on bleeding complication (A) and in-hospital mortality (B); Egger (C) and Begg (D) tests for In-Hospital Mortality; Egger (E) and Begg (F) tests for bleeding complications.

In leave-one-out sensitivity analyses, the pooled effect estimates remained similar; thus, they did not reveal the exertion of any influence on the bleeding complications and in-hospital mortality of individual trials, which confirmed the robustness of the results (Figure 8A,B). Publication bias was evaluated by using Begg’s and Egger’s tests (Figure 8C–F). No evidence of substantial publication bias existed in this meta-analysis on bleeding complications (Egger test, Pr > |z| = 0.306; Begg test, *p* > 0.283). Similar findings were observed for hospital mortality, as indicated by Begg’s test (Pr > |z| = 0.348) and Egger’s test (*p* > 0.340).

### Quality of evidence assessment (GRADE)

In accordance with the GRADE approach, quality assessment for the pooled results of hospital mortality and bleeding complications started at the “LOW” level quality of evidence after downgrading owing to the observational design of most studies and the poor quality of RCTs. The overall quality of evidence ranged from low to very low. As shown in Table 3, Additional Files 4 and 5, a summary of the quality of evidence according to the outcome was depicted.

## Discussion

During the COVID-19 pandemic, a proportion of patients requiring ICU treatment can evolve into severe MODS in the advanced stages of the illness. These patients need different blood purification therapies to prevent organ damage from the existing risk factors and to protect the host against further pathogenic insults [3]. It should be noted that patients undergoing BPT usually carry a high risk of bleeding or suffer from active bleeding. Disseminated intravascular coagulation, a fatal coagulation disorder caused by infective conditions (e.g., severe infection and sepsis) and noninfective conditions (i.e., trauma and hypoxic states), needs to be controlled [37]. Excessive anticoagulation may lead to life-threatening bleeding complications, whereas insufficient anticoagulation can result in poor filter life, treatment interruption and additional costs [38]. Thus, clinicians should take the bleeding risks and benefits of anticoagulants into consideration when choosing safe and effective anticoagulants for their patients. Recently, NM has been used as a clinical therapy for COVID-19, as it can be used for disseminated intravascular coagulation and effectively suppresses MERS-CoV S protein-mediated membrane fusion [11,12]. However, systematic research on NM as a treatment for patients undergoing BPT is limited. In this study, for the first time, we identified all published studies comparing agents with NM for BPT in critically ill patients to assess its effect and safety regarding bleeding complications.

For COVID-19 patients undergoing BPT, the choice of anticoagulants is still based on previous experience. Our meta-analysis demonstrated that CT interventions markedly increased the risk ratio of bleeding complications by 45% (RR =1.45, CI: [1.08, 1.94], *p* = 0.01; *I*^2^ = 44, [fixed-effect model]). UFH, as the most frequently used anticoagulant, has been recommended for severe COVID-19 patients during hemodialysis [39]. Nevertheless, according to Makino S [35] and Ohtake Y [30], the application of UFH confers a higher incidence of bleeding complications than the application of NM. In contrast, Uchino *et al.* reported that bleeding complications occurred in 2% of patients in whom UFH was used and in 3.3% of patients in the NM group [31]. However, it should be noted that the physiopathology of hemodialysis patients with COVID-19 is different from that of common hemodialysis patients. Previous studies have demonstrated that COVID-19 may lead to blood coagulation parameter abnormalities [40]. Infected patients often have thrombocytopenia and sometimes present gingival bleeding, hemorrhinia, or ecchymosis on the skin as their first symptom [41]. Heparin may complicate the control and administration of BPT; for example, the adverse consequences of HIT, which aggravates coagulation dysfunction and requires constant monitoring, can be lethal. Additionally, the late-onset thrombocytopenia of COVID-19 patients makes the contraindications of heparin more difficult to recognize. Low-molecular-weight heparin (LMWH) exerts an anticoagulant effect primarily *via* anti-Xa/anti-IIa activity [42]. Compared with those of UFH, the pharmacokinetics of LMWH is more predictable as a result of the lower nonspecific binding to plasma proteins [43]. Thus, LMWH may have a favorable bleeding complication profile. Nonetheless, in our subgroup analysis, the bleeding complication rate of hemodialysis patients was significantly higher in the LMWH group than in the NM group (RR = 4.58, 95% CI: [1.34, 15.91], *p* = 0.02; *I*^2^ = 0%, *p* = 0.54). LMWH is derived from heparin and may also lead to HIT. A recent meta-analysis showed that the risk of HIT with LMWH and UFH use was 0.2% and 2.6%, respectively [44]. In addition, neither UFH nor LMWH was clearly proven to be superior to NM in our research.

The current guidelines also recommend that BPT proceed without anticoagulation in patients with high bleeding risk [45]. However, it may represent a significant escalation in the cost of their support as more filters are required. The pooled results from our meta-analysis demonstrated that the filter lifespan was significantly decreased in the other groups compared to the NM group (pooled difference in means = −10.59, 95% CI: [−15.45, −5.72], *p* < 0.0001). Furthermore, no significant difference in the risk of bleeding complications was observed between the NM and NA groups (RR = 0.97, *p* = 0.95; *I*^2^ = 0%). With the exponential surge in COVID-19 patients worldwide, increased resources are urgently needed for the unprecedented increase in hemodialysis patients, although they are in short supply [46]. Obviously, a lack of anticoagulant regimens may exacerbate the burden of medical resources. NM can prolong the filter lifespan without an increased risk of bleeding, making the use of resources more efficient and safer.

In the 2012 Kidney Disease Improving Global Outcome (KDIGO) Clinical Practice Guidelines, regional citrate anticoagulation is recommended internationally for acute kidney injury (AKI) patients with bleeding tendencies [45], but the conclusion was drawn from several clinical studies showing the advantages of citrate in the aspect of lower risk of circuit loss, filter failure and bleeding in comparison to heparin [47–49]. Comparative studies on the treatment of NM and citrate for BPT patients are lacking; only one study presented available data in our meta-analysis, and no significant difference in bleeding risk was found between the NM and citrate groups [30]. Furthermore, critically ill patients who undergo BPT with citrate usually have liver dysfunction or other conditions that result in impaired citrate metabolism [50]. Citrate accumulation might cause metabolic complications such as acid-base imbalance, electrolyte abnormalities, hypotension and arrhythmia, while these potential side effects did not exist in anticoagulation with NM [51].

The effect of NM on the mortality of patients undergoing BPT remains controversial. A recent study has shown that critically ill patients undergoing CRRT with NM exhibited no significant differences in transfusion, mortality and survival compared with those who received no anticoagulants [28]. The results from an RCT study with a small sample size (55 patients) showed a trend toward decreased in-hospital mortality in patients who underwent CRRT with NM compared to the no anticoagulant group (47.2% versus 52.8%, *p* = 0.054) [29]. Another previous study indicated that the application of NM observably decreased in-hospital and ICU mortalities in patients with sepsis undergoing BPT compared to the conventional therapy group [36]. In this study, our results showed that the in-hospital mortality rate of patients undergoing BPT with NM ranged from 32% to 66.7%, and the pool rate was 44.6%, which was consistent with a previous study [52]. Furthermore, pooled results showed that the rate of in-hospital mortality in the NM group was significantly decreased compared with that in the CT group. In the subgroup analysis, a significant decrease in the in-hospital mortality rate was observed in the NM group compared with the NA group (RR = 1.31, *p* = 0.002). These data demonstrated the significant effect of NM administration on improving survival outcomes in critically ill patients who underwent blood purification treatment. We speculated that this result might be attributed to the lengthened filter lifespan, more effective fluid removal and clearance, and lower risk of bleeding complications induced by NM administration [29]. Given the trend in the *P* values for in-hospital mortality (RR = 1.19, *p* = 0.05) in the CT group under a random-effects model, further study with a larger sample size might provide statistically significant and more reliable results. Multicenter studies including more participants are needed in the future.

The ideal anticoagulant strategy should include convenient and efficient implementation and monitoring techniques and a beneficial anticoagulant with few side effects. Our meta-analysis showed that NM is safe and effective for BPT and provides substantial benefits to filter lifespan and survival outcomes without increasing bleeding risk. The common adverse effect of NM is hyperkalemia, while precise solute control makes it less likely to occur during hemodialysis [53]. Few severe adverse effects of NM were reported, and there is no contraindication, even in patients with a risk of bleeding. In the present meta-analysis, none of the patients experienced severe adverse effects (e.g., severe anaphylaxis, eosinophilia, agranulocytosis and bone marrow suppression) associated with NM administration. However, it remains possible that some potential side-effects and allergies were ignored or hided due to complex disease status. Though NM exerts as an ideal anticoagulant, the possibility of allergies to NM should be caused alarm, which can be easily and availably evaluated by a skin reaction test [54]. Since nafamostat mesylate has a short half-life of 5–8 min, clinicians can quickly alter the anticoagulation strategy once it is judged to be ineffective [29]. Furthermore, NM is receiving considerable attention for its ability to suppress SARS-CoV-2 S protein-mediated membrane fusion and prevent viral entry at a small blood concentration of 30–240 nM [55]. It may be a promising drug effective against COVID-19 by potently blocking the SARS-CoV-2 entry process and treating DIC with enhanced fibrinolysis [56,57]. Other studies reported that a dose of NM (0.06–0.2 mg/kg/hour) in COVID-19 patient treatment can prevent disease progression by controlling immune system, blocking DIC, and preventing virus invasion [58–60]. Hence, administration of NM with a common dose of 10–30 mg/hour might be a better treatment option for BPT in COVID-19 patients. However, COVID-19 patients were frequently accompanied by coagulopathy that required incremental dosage of anticoagulation (e.g., heparin) both to prevent thrombophilia and to keep circuit patency [3]. In this case, an incremental dosage of NM may be required. Although NM differs from heparin in that no side effects of bleeding are seen even when used at anti-DIC doses, monitoring of anticoagulant activity during blood purification with the incremental dosage of NM would be important to avoid preventable bleeding complications and frequent filter clotting in COVID-19 patients. And intra-circuit activated clotting time (ACT), especially post-filter time-weighted average ACT, might be useful for monitoring anticoagulant activity, NM dosage adjustment and the risk of bleeding complications during BPT with NM [30,32,61].

It should be noted that previous studies have demonstrated NM to be adsorbed to the negatively charged dialysis membrane *via* an ionic bond, including polyacrylonitrile (AN69), acrylonitrile and methallyl sulfonate copolymer (AN69-ST) and J-PAN membranes but not polysulfone (PS), Cuprophan (CU), hemophan (HE), cellulose triacetate (CTA) or polymethylmethacrylate (PMMA) membranes during hemodialysis *in vivo* [62–66]. These three membrane dialyzers, especially AN69 and AN69-ST, observably adsorb NM and may affect the management of anticoagulant therapy. Since most of the enrolled studies in the present meta-analysis did not provide detailed information regarding hemofilter material, the beneficial effect of NM anticoagulation might have been influenced in patients applied with AN69, AN69-ST or J-PAN membrane dialyzers. Thus, in dialysis circuits using these membrane dialyzers, administration of additional post-hemofilter doses of NM may be useful for the management of anticoagulant therapy. Finally, NM was still not cheap. Although using 16.5 mg/h of NM cost US$160 per day more than heparin (US$5 per day) in Korea in 2013, it may save money through prolongation of filter survival due to the expensive filter [33]. Furthermore, NM might improve the survival outcomes of critically ill patients who received blood purification treatments without increasing the rate of significant bleeding events, which ultimately reduced the medical cost. Thus, NM may be recommended for BPT in patients with high-risk bleeding tendencies, while UFH or other anticoagulants with lower prices can be used for the low-risk groups.

Several limitations exist in this meta-analysis. Firstly, most of the enrolled studies were retrospective observational studies; therefore, they may not be representative, and the risk of recall bias may be higher. Secondly, only one included study was a multicenter trial, and the sample size in each study was relatively small, which may have insufficient strength to evaluate the differences and limited the interpretation of our pooled result. Thirdly, we failed to combine filter lifespan-related outcomes in some studies for analysis as they were calculated in different ways that provide different data formats [32–34]. However, the findings of those studies also revealed the same trend that filter life can be increased by the use of NM. Fourthly, only one study presented available data in the present meta-analysis that showed no significant difference in bleeding risk between the NM and citrate groups [12]. Thus, more studies with NM and citrate are urgently required for comparison of the effectiveness and safety of anticoagulation. Fifthly, the present data did not include COVID-19 patients. Further RCTs and systematic analysis are also required for the evaluation of NM in the treatment of COVID-19. Besides, for studies in which this information was presented, there were no differences among studies in the way that disease severity was assessed, and in the dose, timing, and duration of anticoagulant treatment.

## Conclusions

In the current COVID-19 pandemic phase, health resources are being consumed more rapidly than ever before; thus, optimizing filter lifespan and performance efficiency in blood purification treatments is particularly important. NM administration was found to lengthen filter survival time and improve the survival outcomes of critically ill patients who received blood purification treatments without increasing the rate of significant bleeding events. NM can be used as a safe and efficient anticoagulant for blood purification treatments in critically ill patients. In addition, ongoing data collection from studies with larger sample size is still required to elucidate the optimal management procedures of NM to provide superior performance with minimal disruption at the lowest cost.

## Supplementary Material

Supplemental Material

## Data Availability

All data generated or analyzed during this study are included in this published article and its supplementary information files.

## Abbreviations

NM: Nafamostat mesilate
COVID-19: Coronavirus disease 2019
SARS-CoV-2: Severe acute respiratory syndrome coronavirus 2
BPT: Blood purification therapy
CT: Conventional therapy
LMWH: Low-molecular-weight heparin
ICU: Intensive care unit
MODS: Multiple organ dysfunction syndrome
HVHF: High-volume hemofiltration
CPFA: Coupled plasma filtration adsorption
HCO: High-cutoff
UFH: Unfractionated heparin
HIT: Heparin-induced thrombocytopenia
C: Citrate
NA: Anticoagulant-free
CRRT: Continuous renalreplacement therapy
RRT: Renal replacement therapy
CVVH: Continuous venovenous hemofiltration
CVVHD: Continuous venovenous hemodialysis
PE: Plasma exchange
CVVHDF: Continuous venovenous hemodiafiltration
CAVHD: Continuous arteriovenous hemodialysis
PMX-DH: Polymyxin B-immobilized hemoperfusion
AKI: Acute kidney injury

## References

[1] Hodgson SH, Mansatta K, Mallett G, et al. What defines an efficacious COVID-19 vaccine? A review of the challenges assessing the clinical efficacy of vaccines against SARS-CoV-2. Lancet Infect Dis. 2021;21(2):e26–e35. 33125914 10.1016/S1473-3099(20)30773-8 PMC7837315

[2] Tan E, Song J, Deane AM, et al. Global impact of coronavirus disease 2019 infection requiring admission to the ICU: a systematic review and meta-analysis. Chest. 2021;159(2):524–536. 33069725 10.1016/j.chest.2020.10.014 PMC7557272

[3] Ronco C, Bagshaw SM, Bellomo R, et al. Extracorporeal blood purification and organ support in the critically ill patient during COVID-19 pandemic: expert review and recommendation. Blood Purif. 2021;50(1):17–27. 32454500 10.1159/000508125 PMC7270067

[4] Villa G, Romagnoli S, De Rosa S, et al. Blood purification therapy with a hemodiafilter featuring enhanced adsorptive properties for cytokine removal in patients presenting COVID-19: a pilot study. Crit Care. 2020;24(1):605. 33046113 10.1186/s13054-020-03322-6 PMC7549343

[5] Ma J, Xia P, Zhou Y, et al. Potential effect of blood purification therapy in reducing cytokine storm as a late complication of critically ill COVID-19. Clin Immunol. 2020;214:108408. 32247038 10.1016/j.clim.2020.108408 PMC7118642

[6] Monard C, Rimmelé T, Ronco C. Extracorporeal blood purification therapies for sepsis. Blood Purif. 2019;47(Suppl 3):1–14. 10.1159/000499520 30974444

[7] van de Wetering J, Westendorp RG, van der Hoeven JG, et al. Heparin use in continuous renal replacement procedures: the struggle between filter coagulation and patient hemorrhage. J Am Soc Nephrol. 1996;7(1):145–150. 8808122 10.1681/ASN.V71145

[8] Ward DM, Mehta RL. Extracorporeal management of acute renal failure patients at high risk of bleeding. Kidney Int Suppl. 1993;41:S237–S44. 8320930

[9] Guan WJ, Ni ZY, Hu Y, et al. Clinical characteristics of coronavirus disease 2019 in China. N Engl J Med. 2020;382(18):1708–1720. 32109013 10.1056/NEJMoa2002032 PMC7092819

[10] Chen W, Li Z, Yang B, et al. Delayed-phase thrombocytopenia in patients with coronavirus disease 2019 (COVID-19). Br J Haematol. 2020;190(2):179–184. 32453877 10.1111/bjh.16885 PMC7283673

[11] Yamamoto M, Kiso M, Sakai-Tagawa Y, et al. The anticoagulant nafamostat potently inhibits SARS-CoV-2 S protein-mediated fusion in a cell fusion assay system and viral infection in vitro in a cell-type-dependent manner. Viruses. 2020;12(6):629. 10.3390/v12060629 PMC7354595 32532094

[12] Hoffmann M, Schroeder S, Kleine-Weber H, et al. Nafamostat mesylate blocks activation of SARS-CoV-2: new treatment option for COVID-19. Antimicrob Agents Chemother. 2020;64(6):e00754–20. 32312781 10.1128/AAC.00754-20 PMC7269515

[13] Tsukagoshi S. [Pharmacokinetics studies of nafamostat mesilate (FUT), a synthetic protease inhibitor, which has been used for the treatments of DIC and acute pancreatitis, and as an anticoagulant in extracorporeal circulation]. Gan to Kagaku Ryoho. 2000;27(5):767–774. 10832450

[14] Han SJ, Kim HS, Kim KI, et al. Use of nafamostat mesilate as an anticoagulant during extracorporeal membrane oxygenation. J Korean Med Sci. 2011;26(7):945–950. 21738350 10.3346/jkms.2011.26.7.945 PMC3124727

[15] Gutiérrez López de Ocáriz X, Castro Quismondo N, Vera Guerrero E, et al. Thrombosis and antiphospholipid antibodies in patients with SARS-COV-2 infection (COVID-19). Int J Lab Hematol. 2020;42(6):e280–e282. 32851784 10.1111/ijlh.13320 PMC7461094

[16] Arimura T, Abe M, Shiga H, et al. Clinical study of blood purification therapy in critical care in Japan: results from the survey research of the Japan society for blood purification in critical care in 2013. J Artif Organs. 2017;20(3):244–251. 28600615 10.1007/s10047-017-0968-3

[17] Park II, Choi MJ, Yoon JW, et al. Saline versus nafamostat mesilate anticoagulation for continuous veno-venous hemofiltration (CVVH) in patients at high risk of bleeding: a prospective study. Korean J Nephrol. 2009;28(3):205–210.

[18] Liberati A, Altman DG, Tetzlaff J, et al. The PRISMA statement for reporting systematic reviews and meta-analyses of studies that evaluate health care interventions: explanation and elaboration. PLOS Med. 2009;6(7):e1000100. 19621070 10.1371/journal.pmed.1000100 PMC2707010

[19] Higgins JP, Thomas J, Chandler J, et al. Cochrane handbook for systematic reviews of interventions version 6.1. Cochrane, 2020. Available from www.training.cochrane.org/handbook.

[20] National Institutes of Health. National Heart Lung, and Blood Institute. Quality assessment tool for observational cohort and cross-sectional studies. 2014.

[21] Higgins JP, Thompson SG. Quantifying heterogeneity in a meta-analysis. Stat Med. 2002;21(11):1539–1558. 12111919 10.1002/sim.1186

[22] Guyatt GH, Oxman AD, Vist GE, et al. GRADE: an emerging consensus on rating quality of evidence and strength of recommendations. BMJ. 2008;336(7650):924–926. 18436948 10.1136/bmj.39489.470347.AD PMC2335261

[23] Kim HC, Han SY, Kim HK, et al. A multi-center phase III clinical trial to assess the influence to bleeding and anticoagulant effect of nafamostat mesilate (futhan) in hemodialysis patients with high bleeding-risk. Korean J Nephrol. 2004;23(6):920–926.

[24] Akizawa T, Kitaoka T, Sato M, et al. Comparative clinical trial of regional anticoagulation for hemodialysis. ASAIO Trans. 1988;34(3):176–178. 3058171

[25] Nakae H, Igarashi T, Tajimi K. The dose of nafamostat mesilate during plasma exchange with continuous hemodiafiltration in the series-parallel circuit. Ther Apher Dial. 2006;10(3):233–236. 16817786 10.1111/j.1744-9987.2006.00374.x

[26] Inose K, Ono K, Tsutida A, et al. Active inhibitory effect of nafamostat mesylate against the elevation of plasma myeloperoxidase during hemodialysis. Nephron. 1997;75(4):420–425. 9127328 10.1159/000189579

[27] Kim EY, Lee YK, Lee SM, et al. Low-dose nafamostat mesilate in hemodialysis patients at high bleeding risk. Korean J Nephrol. 2011;30(1):61–66.

[28] Lee YK, Lee HW, Choi KH, et al. Ability of nafamostat mesilate to prolong filter patency during continuous renal replacement therapy in patients at high risk of bleeding: a randomized controlled study. PLOS One. 2014;9(10):e108737. 25302581 10.1371/journal.pone.0108737 PMC4193755

[29] Choi JY, Kang YJ, Jang HM, et al. Nafamostat mesilate as an anticoagulant during continuous renal replacement therapy in patients with high bleeding risk: a randomized clinical trial. Medicine. 2015;94(52):e2392. 26717390 10.1097/MD.0000000000002392 PMC5291631

[30] Ohtake Y, Hirasawa H, Sugai T, et al. Nafamostat mesylate as anticoagulant in continuous hemofiltration and continuous hemodiafiltration. Contrib Nephrol. 1991;93:215–217. 1666354 10.1159/000420222

[31] Uchino S, Bellomo R, Morimatsu H, et al. Continuous renal replacement therapy: a worldwide practice survey. The beginning and ending supportive therapy for the kidney (B.E.S.T. kidney) investigators. Intensive Care Med. 2007;33(9):1563–1570. 17594074 10.1007/s00134-007-0754-4

[32] Baek NN, Jang HR, Huh W, et al. The role of nafamostat mesylate in continuous renal replacement therapy among patients at high risk of bleeding. Ren Fail. 2012;34(3):279–285. 22251267 10.3109/0886022X.2011.647293

[33] Hwang SD, Hyun YK, Moon SJ, et al. Nafamostat mesilate for anticoagulation in continuous renal replacement therapy. Int J Artif Organs. 2013;36(3):208–216. 23404639 10.5301/IJAO.5000191

[34] Lee ST, Cho H. The use of nafamostat mesilate as an anticoagulant during continuous renal replacement therapy for children with a high risk of bleeding. Childhood Kidney Dis. 2014;18(2):98–105.

[35] Makino S, Egi M, Kita H, et al. Comparison of nafamostat mesilate and unfractionated heparin as anticoagulants during continuous renal replacement therapy. Int J Artif Organs. 2016;39(1):16–21. 26868216 10.5301/ijao.5000465

[36] Kamijo H, Mochizuki K, Nakamura Y, et al. Nafamostat mesylate improved survival outcomes of sepsis patients who underwent blood purification: a nationwide registry study in Japan. JCM. 2020;9(8):2629. 10.3390/jcm9082629 PMC7464767 32823637

[37] Levi M, Scully M. How I treat disseminated intravascular coagulation. Blood. 2018;131(8):845–854. 29255070 10.1182/blood-2017-10-804096

[38] Pannu N, Gibney RN. Renal replacement therapy in the intensive care unit. Ther Clin Risk Manag. 2005;1(2):141–150. 18360553 10.2147/tcrm.1.2.141.62908 PMC1661614

[39] Song JC, Wang G, Zhang W, et al. Chinese expert consensus on diagnosis and treatment of coagulation dysfunction in COVID-19. Mil Med Res. 2020;7(1):19. 32307014 10.1186/s40779-020-00247-7 PMC7167301

[40] Araya S, Mamo MA, Tsegay YG, et al. Blood coagulation parameter abnormalities in hospitalized patients with confirmed COVID-19 in Ethiopia. PLOS ONE. 2021;16(6):e0252939. 34153056 10.1371/journal.pone.0252939 PMC8216564

[41] Ahmed MZ, Khakwani M, Venkatadasari I, et al. Thrombocytopenia as an initial manifestation of COVID-19; case series and literature review. Br J Haematol. 2020;189(6):1057–1058. 32369609 10.1111/bjh.16769

[42] Hao C, Sun M, Wang H, et al. Low molecular weight heparins and their clinical applications. Prog Mol Biol Transl Sci. 2019;163:21–39. 31030749 10.1016/bs.pmbts.2019.02.003

[43] Hirsh J, Warkentin TE, Shaughnessy SG, et al. Heparin and low-molecular-weight heparin: mechanisms of action, pharmacokinetics, dosing, monitoring, efficacy, and safety. Chest. 2001;119(1 Suppl):64S–94S. 11157643 10.1378/chest.119.1_suppl.64s

[44] Martel N, Lee J, Wells PS. Risk for heparin-induced thrombocytopenia with unfractionated and low-molecular-weight heparin thromboprophylaxis: a meta-analysis. Blood. 2005;106(8):2710–2715. 15985543 10.1182/blood-2005-04-1546

[45] Kellum JA, Lameire N, Aspelin P, et al. Kidney disease: improving global outcomes (KDIGO) acute kidney injury work group. KDIGO clinical practice guideline for acute kidney injury. Kidney Int Suppl. 2012;2(1):1–138.

[46] Reddy YNV, Walensky RP, Mendu ML, et al. Estimating shortages in capacity to deliver continuous kidney replacement therapy during the COVID-19 pandemic in the United States. Am J Kidney Dis. 2020;76(5):696–709.e1. 32730812 10.1053/j.ajkd.2020.07.005 PMC7385068

[47] Liu C, Mao Z, Kang H, et al. Regional citrate versus heparin anticoagulation for continuous renal replacement therapy in critically ill patients: a meta-analysis with trial sequential analysis of randomized controlled trials. Crit Care. 2016;20(1):144. 27176622 10.1186/s13054-016-1299-0 PMC4866420

[48] Stucker F, Ponte B, Tataw J, et al. Efficacy and safety of citrate-based anticoagulation compared to heparin in patients with acute kidney injury requiring continuous renal replacement therapy: a randomized controlled trial. Crit Care. 2015;19(1):91. 25881975 10.1186/s13054-015-0822-z PMC4364313

[49] Oudemans-van Straaten HM, Ostermann M. Bench-to-bedside review: citrate for continuous renal replacement therapy, from science to practice. Crit Care. 2012;16(6):249. 23216871 10.1186/cc11645 PMC3672558

[50] Klingele M, Stadler T, Fliser D, et al. Long-term continuous renal replacement therapy and anticoagulation with citrate in critically ill patients with severe liver dysfunction. Crit Care. 2017;21(1):294. 29187232 10.1186/s13054-017-1870-3 PMC5707786

[51] Okuda A, Uchino S, Shibasaki T, et al. Experience of the continuous hemodialysis with citrate anticoagulation. J Japanese Soc Intensive Care Med. 2013;20(4):653–654.

[52] Zhou F, Peng Z, Murugan R, et al. Blood purification and mortality in sepsis: a meta-analysis of randomized trials. Crit Care Med. 2013;41(9):2209–2220. 23860248 10.1097/CCM.0b013e31828cf412 PMC3758418

[53] Muto S, Imai M, Asano Y. Mechanisms of hyperkalemia caused by nafamostat mesilate. Gen Pharmacol. 1995;26(8):1627–1632. 8745149 10.1016/0306-3623(95)00072-0

[54] Higuchi N, Yamazaki H, Kikuchi H, et al. Anaphylactoid reaction induced by a protease inhibitor, nafamostat mesilate, following nine administrations in a hemodialysis patient. Nephron. 2000;86(3):400–401. 11096324 10.1159/000045822

[55] Yamamoto M, Kiso M, Sakai-Tagawa Y, et al. The anticoagulant nafamostat potently inhibits SARS-CoV-2 S protein-mediated fusion in a cell fusion assay system and viral infection in vitro in a cell-type-dependent manner. Viruses. 2020;12(6):629. 10.3390/v12060629 PMC7354595 32532094

[56] Takahashi W, Yoneda T, Koba H, et al. Potential mechanisms of nafamostat therapy for severe COVID-19 pneumonia with disseminated intravascular coagulation. Int J Infect Dis. 2021;102:529–531. 33157292 10.1016/j.ijid.2020.10.093 PMC7607231

[57] Asakura H, Ogawa H. Potential of heparin and nafamostat combination therapy for COVID-19. J Thromb Haemost. 2020;18(6):1521–1522. 32302456 10.1111/jth.14858 PMC9906352

[58] Jang S, Rhee JY. Three cases of treatment with nafamostat in elderly patients with COVID-19 pneumonia who need oxygen therapy. Int J Infect Dis. 2020;96:500–502. 32470602 10.1016/j.ijid.2020.05.072 PMC7250091

[59] Doi K, Ikeda M, Hayase N, et al. Nafamostat mesylate treatment in combination with favipiravir for patients critically ill with covid-19: a case series. Crit Care. 2020;24(1):392. 32620147 10.1186/s13054-020-03078-z PMC7332736

[60] Okajima M, Takahashi Y, Kaji T, et al. Nafamostat mesylate-induced hyperkalemia in critically ill patients with COVID-19: four case reports. World J Clin Cases. 2020;8(21):5320–5325. 33269265 10.12998/wjcc.v8.i21.5320 PMC7674713

[61] Miyatake Y, Makino S, Kubota K, et al. Association between intra-circuit activated clotting time and incidence of bleeding complications during continuous renal replacement therapy using nafamostat mesilate: a retrospective pilot observational study. Kobe J Med Sci. 2017;63(1):E30–E36. PMC5824928 29434171

[62] Inagaki O, Nishian Y, Iwaki R, et al. Adsorption of nafamostat mesilate by hemodialysis membranes. Artif Organs. 1992;16(6):553–558. 1482323 10.1111/j.1525-1594.1992.tb00551.x

[63] Hirayama T, Nosaka N, Okawa Y, et al. AN69ST membranes adsorb nafamostat mesylate and affect the management of anticoagulant therapy: a retrospective study. J Intensive Care. 2017;5:46. 28729905 10.1186/s40560-017-0244-x PMC5516335

[64] Shiraishi Y, Okajima M, Sai Y, et al. Elimination of teicoplanin by adsorption to the filter membrane during haemodiafiltration: screening experiments for linezolid, teicoplanin and vancomycin followed by in vitro haemodiafiltration models for teicoplanin. Anaesth Intensive Care. 2012;40(3):442–449. 22577909 10.1177/0310057X1204000309

[65] Nakamura Y, Hara S, Hatomoto H, et al. Adsorption of nafamostat mesilate on AN69ST membranes: a single-center retrospective and in vitro study. Ther Apher Dial. 2017;21(6):620–627. 28960755 10.1111/1744-9987.12587

[66] Renaux JL, Thomas M, Crost T, et al. Activation of the kallikrein-kinin system in hemodialysis: role of membrane electronegativity, blood dilution, and pH. Kidney Int. 1999;55(3):1097–1103. 10027949 10.1046/j.1523-1755.1999.0550031097.x

